# Iron-Based Shape Memory Alloys in Construction: Research, Applications and Opportunities

**DOI:** 10.3390/ma15051723

**Published:** 2022-02-25

**Authors:** Zhe-Xi Zhang, Jie Zhang, Honglei Wu, Yuezhen Ji, Dheeraj D. Kumar

**Affiliations:** 1 Department of Structural Engineering, College of Civil Engineering, Tongji University, Shanghai 200092, China; zzx888@tongji.edu.cn (Z.-X.Z.); 2032316@tongji.edu.cn (J.Z.); 1832499@tongji.edu.cn (Y.J.); 2093369@tongji.edu.cn (D.D.K.); 2 Tongji Architectural Design (Group) Co., Ltd., Shanghai 200092, China; 3 China Railway ERYUAN Engineering (Group) Co., Ltd., Chengdu 610031, China

**Keywords:** iron-based shape memory alloy (Fe-SMA), shape memory effect, martensitic transformation, prestressing, low cycle fatigue, seismic, damping

## Abstract

As a promising candidate in the construction industry, iron-based shape memory alloy (Fe-SMA) has attracted lots of attention in the engineering and metallography communities because of its foreseeable benefits including corrosion resistance, shape recovery capability, excellent plastic deformability, and outstanding fatigue resistance. Pilot applications have proved the feasibility of Fe-SMA as a highly efficient functional material in the construction sector. This paper provides a review of recent developments in research and design practice related to Fe-SMA. The basic mechanical properties are presented and compared with conventional structural steel, and some necessary explanations are given on the metallographic transformation mechanism. Newly emerged applications, such as Fe-SMA-based prestressing/strengthening techniques and seismic-resistant components/devices, are discussed. It is believed that Fe-SMA offers a wide range of applications in the construction industry but there still remains problems to be addressed and areas to be further explored. Some research needs at material-level, component-level, and system-level are highlighted in this paper. With the systematic information provided, this paper not only benefits professionals and researchers who have been working in this area for a long time and wanting to gain an in-depth understanding of the state-of-the-art, but also helps enlighten a wider audience intending to get acquainted with this exciting topic.

## 1. Introduction

Iron-based shape memory alloy (Fe-SMA, especially referring to Fe-Mn-Si class shape memory alloy) possesses shape memory effect (SME) [1,2], outstanding low-cycle fatigue (LCF) resistance [3], and some other desirable characteristics by which the material has proven its potential in the field of the construction industry. Fe-SMA was traditionally regarded as an ideal material used in fasteners and tie systems, e.g., pipe joints, rail couplings, and crane rail joint plates, where constrained stress is required [4]. The main purpose of using such materials is to simplify the construction process by its SME property which induces prestress conveniently. The so-called SME-induced prestress, also known as ‘recovery stress’, is associated with its unique deformation-induced martensitic transformation and subsequent heating–cooling process, where an approximate stress of 200–400 MPa can be generated.

Apart from its desirable prestressing capability, Fe-SMA also has excellent low-cycle fatigue resistance, which was first recognized by Sawaguchi in 2006 [3]. A series of studies have been carried out on this front and considerable achievements have been made. The engineering community has gained particular confidence with the completion of the 196-m skyscraper ‘JP Tower Nagoya’, where Fe-SMA seismic dampers were first employed in an actual project in seismic-prone cities. These works further expand the application boundary of Fe-SMA and inspire the interests of seismic engineers.

Fe-SMA has some extra benefits. It is reported that the corrosion-resistance of Fe-SMA is close to that of stainless steel due to the addition of Nickel and Chromium elements [5]. This makes Fe-SMA well suited to chloride environments, e.g., coastal/offshore engineering construction. In addition, in contrast to Nitinol (another popular class of SMA) which is less easily produced in large scale because of the demanding metallurgical process [6,7,8], Fe-SMA can be mass produced with conventional metallurgical equipment [9], and, more encouragingly, the cost of the raw materials is inherently low [10]. This facilitates practical use of Fe-SMA in the civil engineering sector, where the necessary size of elements/members is often large and the budget is often controlled.

This paper seeks to summarize the recent technological advances in the research and application of Fe-SMA in construction, covering diverse aspects including structural retrofitting and seismic damping. The paper is also keen to highlight the authors’ unique reflections on the present issue and future needs in this field, in addition to the latest solutions. An overview of the basic mechanical properties of Fe-SMA is first presented, followed by the possible application scenarios emerging over the past 10 years. Challenges arising from the application of the new material are described, and where further studies are emphasized that are required to respond to the identified issues. Research needs and new application opportunities of Fe-SMA are also presented. While this paper is of scientific interest to the mechanical and material science community, much emphasis is placed on making the contents easier to learn by civil engineers. Therefore, in most cases, the presentation is structured following the civil engineering custom and terminology.

## 2. Basic Properties

Prior to the introduction of the application of Fe-SMA, it is necessary to have a comprehensive understanding of its basic characteristics. Typical physical properties of Fe-SMA together with other important steel types are listed in Table 1. The Fe-SMA presented in the table is Fe-17Mn-5Si-10Cr-4Ni-1(V,C) (mass%), which is one of the most typical classes. Other classes will be expressed in the form of their compositions when they are discussed.

### 2.1. Metallographic Transformation in Fe-SMA

**Figure 1:**
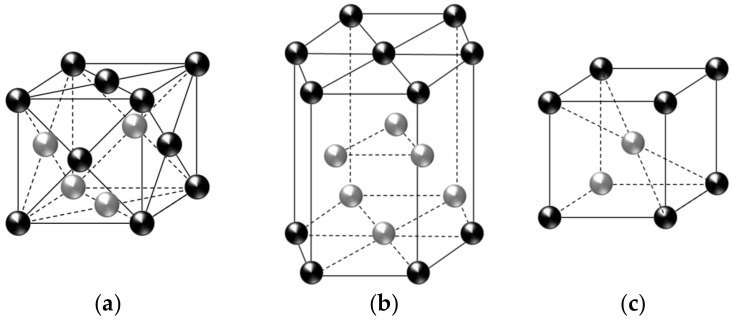
Micro-structure of crystal lattices: (**a**) γ-austenite; (**b**) ε-martensite; (**c**) α’-martensite.

When a metallic substance is subjected to an external force, metallographic transformation process is triggered. At the micro-level, the crystal lattice matrix is rearranged and the atoms move in a particular way. The accumulated movements of the atoms on the microscopic scale directly leads to deformation of the metal on a macroscopic scale [5]. Fe-SMA has three types of metallographic phases, i.e., γ-austenite (face-centered cubic structure, fcc), ε-martensite (hexagonal close-packed structure, hcp), and α’-martensite (body-centered tetragonal structure, bct). Schematic illustrations of these crystal lattices are shown in Figure 1. The metallographic transformation between γ-austenite and ε-martensite (so-called ‘martensitic transformation’) occurs when the material is under an applied force and/or a temperature change, and thus the martensitic transformation of Fe-SMA can be categorized into stress-induced martensitic transformation and thermal-induced martensitic transformation.

**Figure 2:**
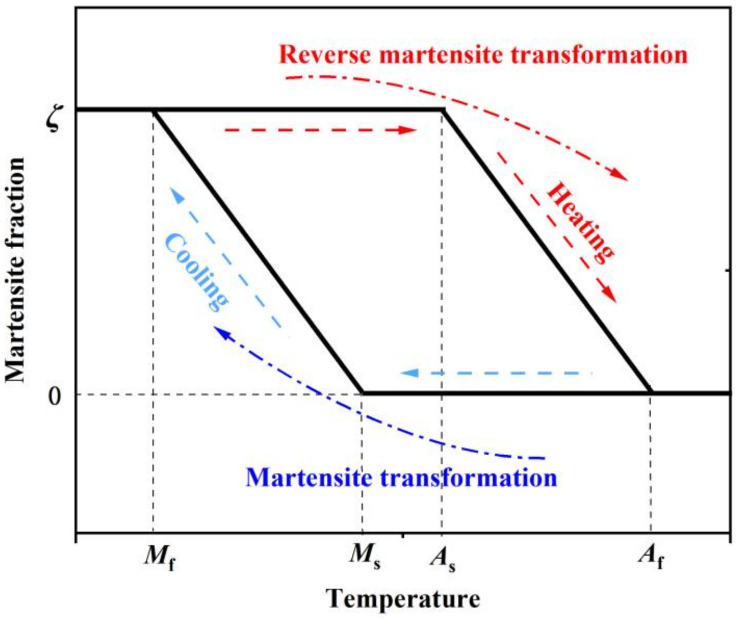
Schematic definition of thermal-induced martensitic transformation of Fe-SMA. ζ denotes the maximum martensite fraction, and 0 < ζ < 1.

The principle of thermal-induced martensitic transformation and its reverse process can be readily understood by the following description. As seen in Figure 2, four typical phase-transformation temperatures, i.e., martensite start temperature (*M*_s_), martensite finish temperature (*M*_f_), austenite start temperature (*A*_s_), and austenite finish temperature (*A*_f_), are representative ones related to the start and finish of martensitic transformation or its reverse process. When the initial temperature is high and it decreases to *M*_s_, the transformation from γ-austenite to ε-martensite starts and then the ε-martensite fraction in Fe-SMA increases gradually. When the temperature drops below *M*_f_, the transformation process is completed and the ε-martensite fraction in Fe-SMA reaches its maximum value (i.e., ζ in Figure 2). For the reverse transformation, when the rising temperature reaches *A*_s_, the ε-martensite starts to transform into the γ-austenite phase, and this process continues until the temperature increases beyond *A*_f_, where the ε-martensite is transformed into γ-austenite completely. Table 2 gives the measured results of the critical phase-transformation temperatures of typical SMAs.

The martensitic transformation of Fe-SMA has two characteristics which directly promote its unique mechanical behavior: (1) The stacking fault energy required for martensitic transformation is low, which makes the martensitic transformation easy to occur [5], (2) The martensitic transformation is a diffusionless phase transformation process (i.e., it creates a new crystal structure without introducing any compositional change) [5], making its reversible martensitic transformation possible. The former gives the basis on why the transformation between γ-austenite and ε-martensite would take place first when Fe-SMA is exposed to external force and the latter explains the basic mechanism of pseudo-elasticity & shape memory effect (this part will be further described in Section 2.3). More detailed descriptions of the martensitic transformation from a perspective of crystallography can be found elsewhere [5,17].

### 2.2. Monotonic Loading Property

**Figure 3:**
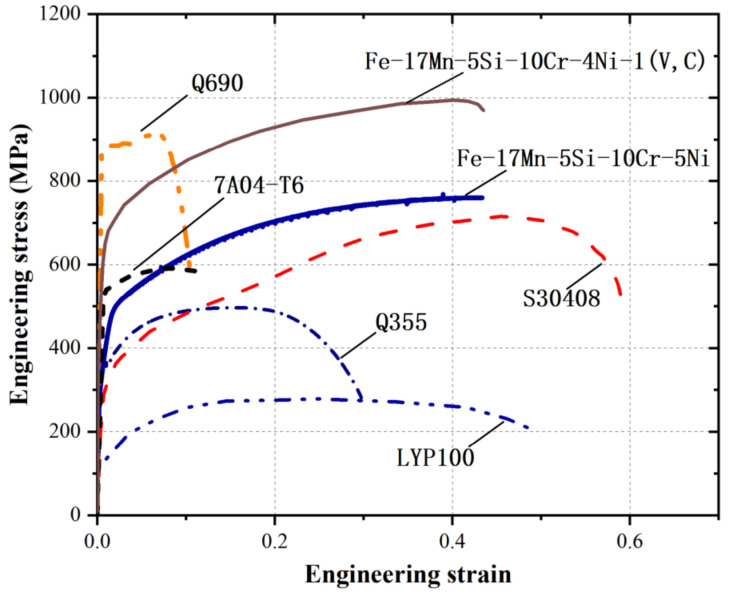
Typical full stress-strain curves of Fe-SMA and other typical structural steels [19,20,21,22,23,24].

**Figure 4:**
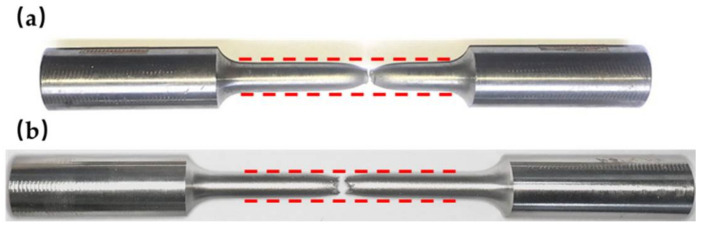
Macroscopic fracture behavior of (**a**) Q355 and (**b**) Fe-SMA under monotonic loading.

Figure 3 compares the typical monotonic stress-strain curves of Fe-SMA with those of other structural steel, i.e., austenitic stainless steel S30408, low point steel LYP100, mild steel Q355, high-strength steel Q690 and aluminum alloy 7A04-T6. The stress-strain responses of Fe-SMAs seem to be similar to that of stainless steel, although the strength of the former is higher. Since there is no yield plateau in Fe-SMA, 0.2% proof stress is considered as an equivalent yield strength. The comparison of the basic mechanical properties between Fe-SMA and other metals is summarized in Table 3. The Young’s moduli of the Fe-SMAs are generally comparable to, and may be slightly lower than, those of steel (except for aluminum alloy). High ultimate strengths (*f*_u_ > 700 MPa) and remarkable ductility are observed in the Fe-SMAs, characteristics which are encouraging for seismic application. The yield to ultimate strength ratio of Fe-SMA is around 0.4, indicating a pronounced strain hardening behavior. Fracture of Fe-SMA occurs soon after reaching the ultimate strength, indicating an inadequate necking process. This feature can be more clearly reflected when compared with conventional structural steel (such as mild steel Q355) where a significant localized shrinkage appears before fracture, as shown in Figure 4. This phenomenon also indicates that the ductility of Fe-SMA is mainly derived from its evenly distributed plasticity rather than localized necking. Apart from these characteristics, Fe-SMA is also reported to possess a higher yield strength at higher strain rates [18], which may make Fe-SMA an ideal material for blast-resistant structures, but no such study is currently available.

**Figure 5:**
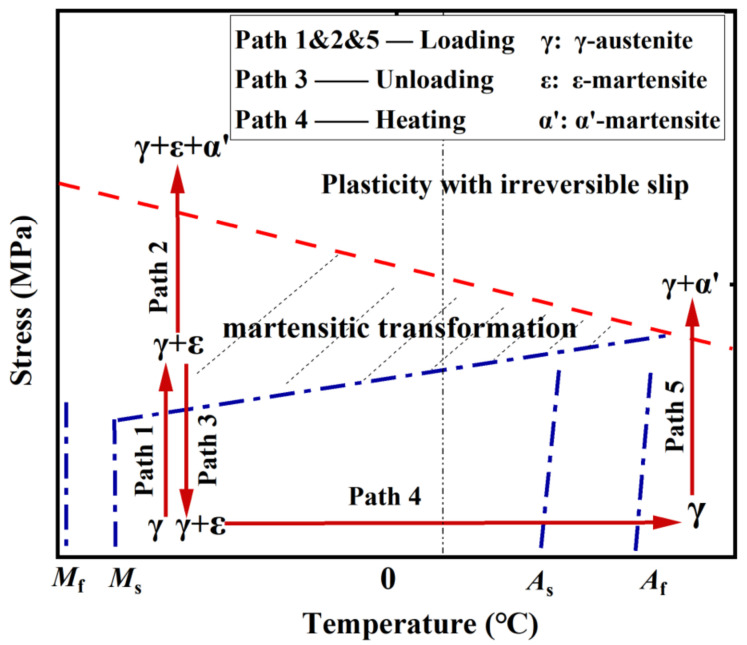
Evolution law of metallographic transformation for Fe-SMA under different thermal-mechanical states.

The evolution law of metallographic transformation during monotonic loading process [29,30,31,32,33,34] can be described as follows: the initial phase of Fe-SMA specimen can be considered pure γ-austenite since it is annealed during manufacturing, a process which is equivalent to the reverse transformation mentioned above. As the stress (or strain) increases, part of the parent γ-austenite gradually transforms into ε-martensite (see Path 1 in Figure 5), and this leads to a deviation of the monotonic stress-strain curve from the linear relationship [5,35]. As the stress further increases (see Path 2 in Figure 5), α’-martensite, whose structure is formed through the lattice expansion of fcc and hcp structures, is discovered in the field of γ-austenite and ε-martensite [5,9]. This transformation process continues until fracture. It is highlighted that when the material is loaded under a high service temperature, the formation of stress-induced ε-martensite may be strongly hindered, and this process is replaced by a direct transformation from parent γ-austenite to α’-martensite (see Path 5 in Figure 5).

### 2.3. Pseudo-Elasticity and Shape Recovery Property

**Figure 6:**
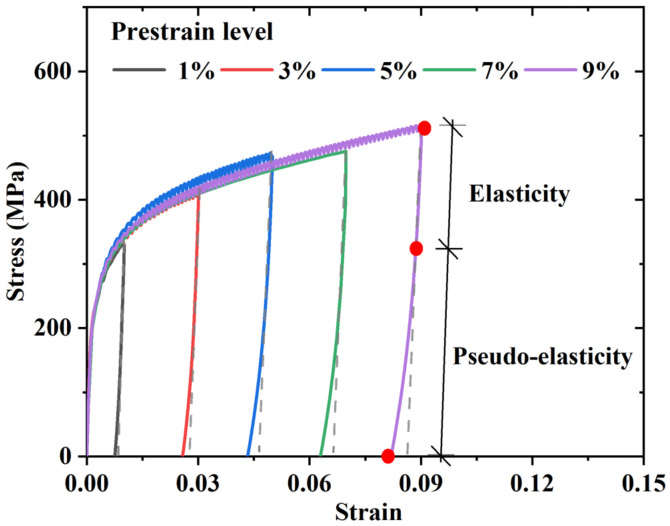
Pseudo-elasticity phenomenon of Fe-SMA.

**Figure 7:**
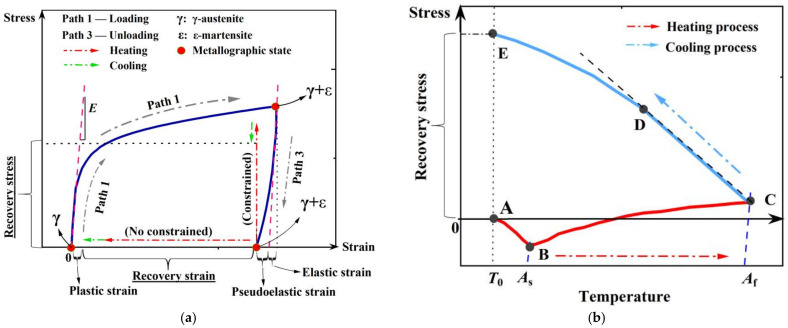
Schematic diagram of (**a**) activation process of shape recovery property, and (**b**) generation of recovery stress.

Hooke’s law holds true for normal steel during both the loading and unloading stages. However, a nonlinear spring-back curve deviating from the linear path exists in Fe-SMA upon unloading (see Figure 6 and Figure 7a). This unique phenomenon is called pseudo-elasticity [36,37,38,39,40,41,42], which is associated with the partial reverse transformation of the previously formed stress-induced ε-martensite. However, due to the limited fraction of stress-induced ε-martensite, the phenomenon of pseudo-elasticity is limited, i.e., much less significant than Nitinol [21,23,43,44]. In any case, the moderate pseudo-elasticity could still benefit residual deformation control during dynamic shakedown, as discussed later in Section 2.4.

Shape recovery property, also known as shape memory effect (SME), is activated by heating the deformed material. The aforementioned thermal-induced ε→γ transformation contributes to the SME of Fe-SMA elements. Considering a Fe-SMA bar which is subjected to axial elongation and subsequently unloaded, residual deformation occurs, like normal steel. If the residual deformation of this bar is constrained, recovery stress (pre-stress) is generated after heating and cooling. Figure 7b illustrates the development of recovery stress (corresponding to path 4 in Figure 5). In the initial heating stage, stress relaxation is observed due to thermal expansion (path A→B). When the temperature reaches *A*_s_, thermal-induced ε→γ transformation is triggered and the recovery stress starts to counteract thermal expansion. This process continues until the temperature increases to the predefined maximum temperature (path B→C). During the cooling stage (e.g., air cooling), a tensile stress is generated due to contraction and thus further recovery stress is induced, which first increases linearly with decreasing temperature (path C→D). As the tensile stress increases to a threshold of the minimum value for triggering the stress-induced martensite transformation, Fe-SMA again enters into the plastic stage. Consequently, a ‘yield’ phenomenon is observed (point D) and the stress-temperature curve becomes nonlinear (path D→E). Point E is the final recovery stress produced in this process. It is noteworthy that the preload level (i.e., path 1 in Figure 5) should be controlled within a moderate range to avoid the formation of α’-martensite, since the reverse transformation process cannot be realized when the material is in the α’-martensite state [9]. In other words, α’-martensite is responsible for the irreversible plasticity, i.e., unrecoverable plastic strain or permanent deformation.

When Fe-SMA is employed as a measure of prestressing, the amount of prestress is the property of most concern to engineers. With the aim of increasing the shape recovery properties, many research groups across the world have been devoted to developing improved production and manufacturing processes, including but not limited to thermal mechanical training, adjusting the chemical compositions and heat treatment [45,46,47,48,49,50,51,52,53,54,55,56,57,58,59,60,61,62,63,64,65,66,67,68,69,70,71]. These efforts greatly enhance the reliability of Fe-SMA as an emerging prestressing strategy. For engineering practice, a satisfactory prestress level can be achieved by pre-loading and heating the Fe-SMA in an appropriate way. Table 4 summarizes the reported recovery stress (*f*_R_) of Fe-SMA generated from different activation conditions. A pre-strain level of 2–4% seems to be an optimum range to achieve a satisfactory recovery stress (300–450 MPa) when the activation temperature is below 200 °C [14,21,41,72]. It is also interesting to find that concurrently applying a higher activation temperature (350 °C) and a larger pre-strain level (6–8%) is beneficial for producing a larger recovery stress (which is almost 30% higher than that activated at 200 °C) [73]. An increase in the activation temperature may cause problems to concrete but is acceptable for steel structures [74]. Instead of applying monotonic pre-strain, researchers have also examined the recovery stress of Fe-SMA upon heating after experiencing tension-compression strain cycles, where the shape recovery capability is decreased [75]. One possible reason is that the compression history worsens the micro-structural environment for the reverse martensitic transformation process.

### 2.4. Cyclic Behavior, Low Cycle Fatigue and Energy Dissipation Capacity

The potential of Fe-SMA as energy-dissipating material was not recognized until 2006, when its stable hysteretic behavior and excellent low cycle fatigue resistance were first identified by Sawaguchi et al. [77]. For seismic application, it is essential to clarify the mechanical behavior of Fe-SMA under cyclic loading [78,79,80,81,82,83,84,85,86,87,88,89,90,91,92,93,94]. In this section, some basic properties of Fe-SMA under cyclic loading are discussed.

#### 2.4.1. Hysteretic Behavior

**Figure 8:**
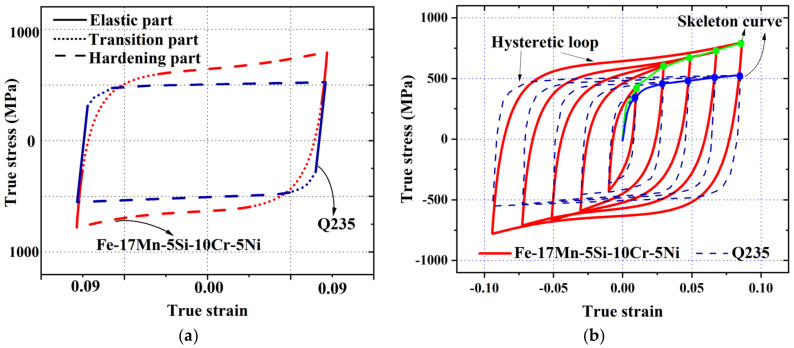
Comparison of (**a**) Decomposed hysteretic loops; and (**b**) Skeleton curves obtained from stabilized hysteretic loops of Fe-17Mn-5Si-10Cr-5Ni and mild steel (Q235).

Obtaining the hysteretic behavior under symmetrical cyclic loading is often a first and standard procedure to understand the basic performance of a member or material during earthquakes [95,96,97,98,99,100]. Figure 8a shows typical stabilized hysteretic loops (taken from half-life cycle response) of Fe-SMA and steel, where the curves are divided into the elastic (linear) part, transition part and hardening part. It is found that the loop shape of the Fe-SMA is slightly narrower than that of the mild steel. An early spring-back phenomenon, which results from the aforementioned pseudo-elasticity, is displayed during the unloading process, leading to a smaller elastic region. In addition, Fe-SMA shows a more obvious strain hardening response, whereas the mild steel shows little hardening with an almost flat stress-strain curve in the hardening part. It is reasonable to deduce that the residual deformation of structures with Fe-SMA-based components would be reduced during earthquake conditions, since the spring-back self-centering trend, together with a relatively large post-yield hardening, could effectively promote a self-centering capability of the system under dynamic shakedown [101,102]. Figure 8b further presents the full incremental stress-strain responses as well as the skeleton curves (obtained from connecting the peak stresses of the hysteretic loops) of Fe-SMA and mild steel.

Researchers have reported that the hysteretic loops of Fe-SMA can quickly saturate regardless of the strain range so that the peak stress is stable until failure [23]. However, according to the findings reported by Rosa et al. [26], a slight cyclic softening behavior (i.e., decrease in peak stress) appears when the strain rate reaches 0.08/s, which is an expected strain rate considering a real earthquake excitation [103]. Further research opportunity exists in comprehensive understanding of the rate effect of the material, especially in the context of seismic application.

**Figure 9:**
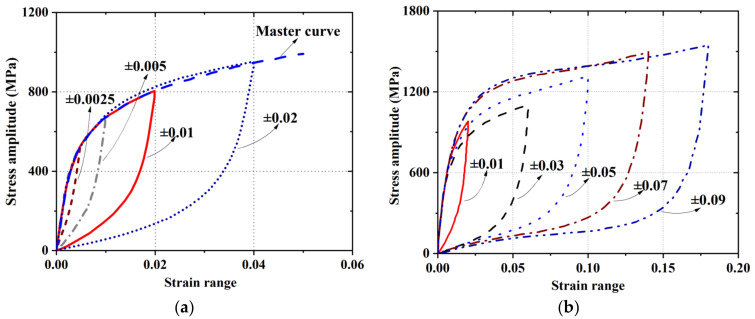
Adaptability of Fe-SMA to Masing behavior under (**a**) small strain ranges [84]; and (**b**) large strain ranges [23].

Figure 9 presents the stabilized hysteretic loops at different strain amplitudes by moving their compressive corners to the same coordinate origin. If the ascending curves of these hysteresis loops coincide, i.e., a master curve can be drawn in accordance with the ascending curves, the material is deemed to possess Masing behavior. Previous research [84] reported that Masing behavior can be observed in Fe-SMA when the strain amplitudes are less than ±2%, as shown in Figure 9a. However, when the strain amplitude increases, the ascending branches of the saturated hysteresis loops deviate from the master curve and represent a non-Masing behavior (Figure 9b). Microstructural observations reveal that the adaptability of Fe-SMA to Masing behavior is related to its micro-deformation patterns. When the strain amplitude is moderate (e.g., not exceeding ±2%), the strain-induced martensitic transformation accompanied by a planar slip of Shockley partial dislocations in austenite is the main deformation mode [84]. However, when the strain amplitude advances to a larger range, the micro-deformation pattern is dominated by the formation of mechanical-twinning [90].

#### 2.4.2. Low Cycle Fatigue (LCF) Behavior

**Figure 10:**
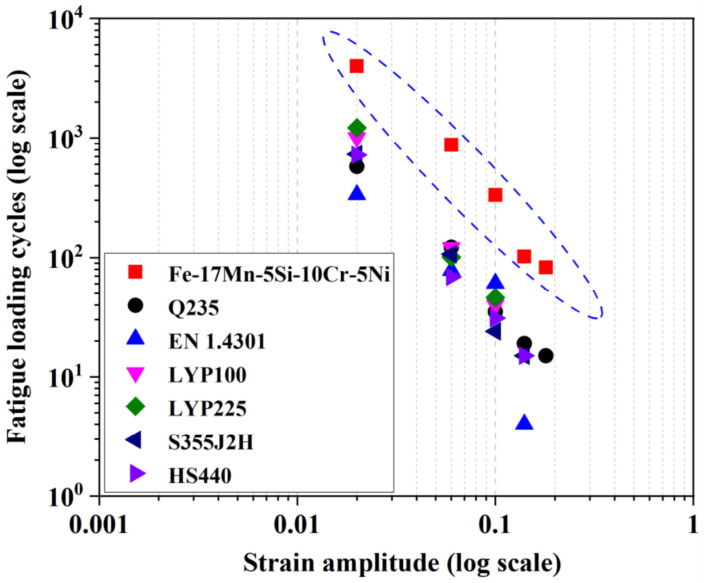
Comparison of fatigue lives between Fe-SMA and other typical metals [23,108,109].

**Figure 11:**
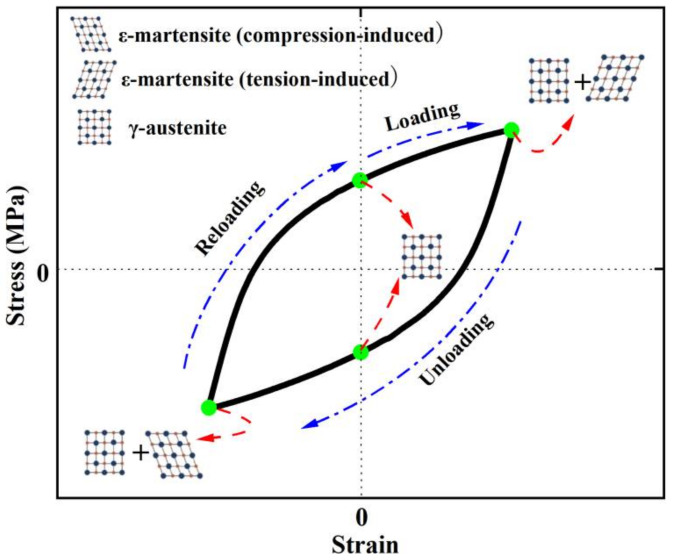
Phase transformation during cyclic tension-compression process for Fe-SMA.

Large-size Nitinol elements are often criticized for their brittle fracture behavior and poor LCF resistance [104,105,106,107]. Figure 10 and Table 5 summarize the LCF lives of some typical steels. It can be seen that Fe-SMA possesses significantly longer LCF life than conventional steels. This remarkable property results from the evolution of cyclically deformation-induced ε-martensitic transformation, in contrast to dislocation-based plasticity with irreversible slip in normal steels. As demonstrated in Figure 11, the parent γ-austenite phase partially transforms into a tension-induced ε-martensite during the loading process. During the unloading stage, the tension-induced martensite gradually diminishes and returns back to γ-austenite. When subjected to compression towards negative strain, compression-induced ε-martensite is generated in the field of γ-austenite [77,78]. The phase transformation of the re-loading stage is similar to that of the aforementioned unloading stage, and the repeated tension-compression cyclic loading process induces alternate formation and disappearance of stress-induced ε-martensite. The repeated phase transformation is beneficial in reducing the internal stress concentration caused by cyclic loading and inhibiting local accumulation of dislocations and hence the initiation and propagation of fatigue cracks [88].

#### 2.4.3. Energy Dissipation

The equivalent viscous damping ratio (EVD), as defined in Equation (1), is considered to evaluate the energy dissipation capacity of Fe-SMA specimens under fully reversed cyclic loading.

(1) $$\mathrm{EVD}=\frac{1}{2\pi }\frac{E_{D}}{E_{S}}$$

**Figure 12:**
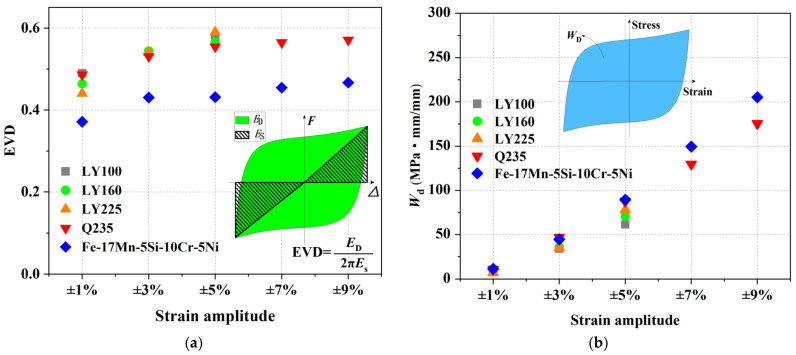
Parameters for evaluating energy dissipation capacity under different strain amplitudes: (**a**) EVDs; and (**b**) Absolute energy dissipation at half-life cycle. Results are re-produced from [23,25].

In the equation, *E_D_* is the area within the inelastic force-displacement response curve, and *E_S_* is the recoverable elastic strain energy stored in an equivalent linear elastic system [110]. The EVDs of different metals under half-life cycle are plotted in Figure 12a. It can be seen that the EVDs of Fe-SMA are lower (decreased by about 20%) than that of conventional steel. This is mainly attributed to its strain hardening behavior, resulting in a narrower hysteresis loop shape. The early spring-back behavior also decreases the *E_S_* of Fe-SMA to some degree. On the other hand, the absolute energy dissipation *W_D_*, which is calculated by the area of the stabilized stress-strain curves, shows that Fe-SMA could be have more energy dissipation, especially at larger strain amplitudes (see Figure 12b). More importantly, due to the excellent LCF resistance, the total energy dissipation (accumulation of *E_D_*) of Fe-SMA is much larger than that of normal steel with the same geometric size.

## 3. Research and Potential Engineering Applications

Early application of Fe-SMA in civil engineering began in the 1990s, where focus was mainly on special connections, such as railway fishplates, crane rail joint plates, and pipe connection devices for tunnel construction [5,9,17,111]. These connections are tightened via the SME of Fe-SMA. The SME-triggered tightening method greatly simplifies the construction/prestressing process.

Recent research and development activities enable a wider use of Fe-SMA in the construction industry. In particular, the research and application of Fe-SMA over the past 10 years can be mainly divided into two fields: (1) SME-induced prestressing technique for repairing and strengthening structures [112,113,114,115,116,117,118,119,120,121,122,123,124,125,126,127,128,129,130,131,132,133,134,135,136,137,138,139], and (2) seismic damping [23,28,75].

### 3.1. Novel Strengthening Solution Based on SME of Fe-SMA

**Figure 13:**
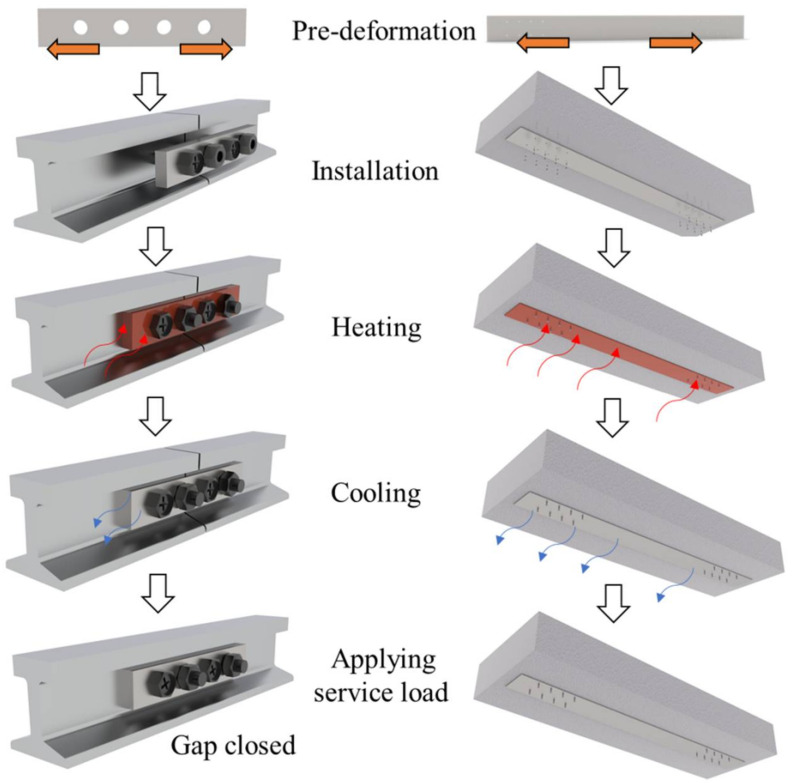
Schematic illustration of procedure for Fe-SMA-based strengthening/connecting solution.

High-cycle fatigue causes crack propagation in structural components, a case which in turn leads to the deterioration of stiffness/strength and shortens the service life of the structure. Practice has proven that through strengthening the cracked or damaged components, the service life can be effectively prolonged. Existing strengthening strategies include using external bonding reinforcing materials or applying prestressing. Overcoming some possible shortcomings such as the difficulty in construction for the traditional reinforcing strategies, a new method utilizing Fe-SMA has received great attention. The main procedure of the Fe-SMA-based strengthening solution is similar to that of the aforementioned SME-triggered tightening method and can be summarized as follows (see Figure 13):

(1) Pre-deform (for most cases, pre-tension) the Fe-SMA elements to a preset strain value or the expected shape;
(2) Connect the pre-deformed Fe-SMA elements to the base components (i.e., those ready for strengthening or connecting);
(3) Apply electric heating (or infrared heating) to the pre-deformed Fe-SMA elements to a preset temperature and keep it for a short period of time to guarantee fully activated SME;
(4) Wait until the Fe-SMA elements cool down to room temperature, and recovery stress is generated;
(5) The structures then return to normal service state.

#### 3.1.1. Strengthening for Reinforced Concrete (RC) Structures

The earliest practical application of Fe-SMA in the field of prestressing can be traced back to 2001 where a bridge in Michigan, United States, experienced fatigue-induced cracking [120]. In this case, Fe-SMA tendons were installed perpendicularly to the shear cracks. After electric heating, a recovery stress of approximately 225 MPa was induced in the Fe-SMA tendons. Field measurements indicated that the generated recovery stress closed the width of the shear cracks to a large extent and the load-carrying capacity of this bridge was effectively recovered.

**Figure 14:**
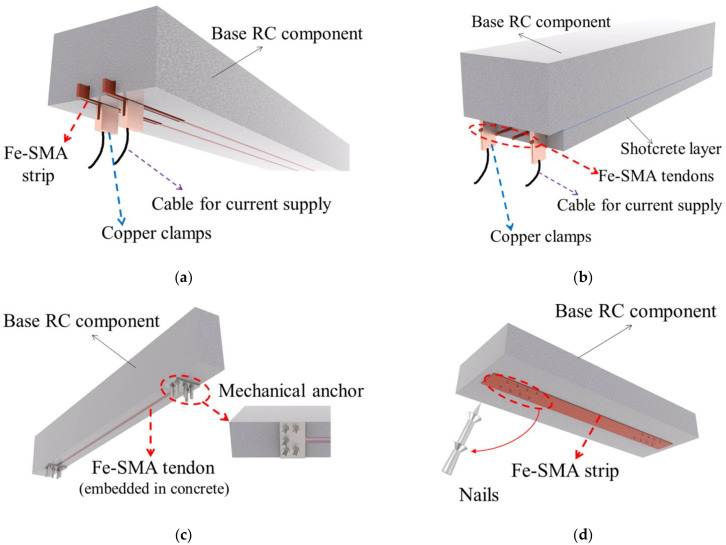
Schematic illustration of different anchoring methods for Fe-SMA tendon/strip strengthening RC components: (**a**) NSMR method; (**b**) shotcrete method; (**c**) NSMR-expansion anchor method; and (**d**) nail-based method.

Since the initial success, laboratory research works have been conducted on concrete structures strengthened by Fe-SMA reinforcement, and suitable anchorage systems have been developed. For example, EMPA (Swiss Federal Laboratories for Materials Science and Technology) proposed an anchorage system for Fe-SMA tendons employed as near-surface mounted reinforcement (NSMR) in RC structures [121,122,123]. As shown in Figure 14a, Fe-SMA prestressing elements are embedded in pre-made grooves and covered with adhesive material such as cement-based mortar herein. Lap-shear experiments have been carried out to clarify the bonding behavior between the Fe-SMA strips and cement-based mortar [122]. Deeper embedment depth and ribbed surface for Fe-SMA strips are recommended for practical application [124]. It is also found that the current design guidelines would underestimate the necessary anchorage length for Fe-SMA bars [125]. The corresponding calculation methods are yet to be available.

With the aim of further simplifying the anchoring process and satisfying the objective of rapid recovery on site, a new anchoring method employing shotcrete is proposed (see Figure 14b) [126]. The previously tensioned Fe-SMA tendons are installed beneath the beam with an additional cementitious layer (shotcrete) sprayed on, covering the Fe-SMA tendons. After sufficient curing, current resistance heating is applied for activation and prestress is induced. Feasibility studies on flexural strengthening [126] and shear strengthening [127] of RC beams with this anchoring method have been conducted. Both the test results revealed that this strengthening system can efficiently increase the flexural/shear-resistant performance of RC beams. At the same time, beam deflections, number of cracks, and the widths of cracks were all reduced.

The flexural behavior of RC beams strengthened by the Fe-SMA NSMR system was investigated in [128], where ribbed Fe-SMA strips were longitudinally embedded at the bottom of the beam. Copper clamps were used to transmit the electric current. After current resistance heating to a target temperature of 160 °C, a permanent prestress of about 200 MPa was created in the Fe-SMA strips. Rojob et al. [129] conducted a comparative experimental study on the effect of strengthening through CFRP strips and Fe-SMA strips, and confirmed that the Fe-SMA strips lead to better ductility of the beam. Rojob et al. [130] further added expansion anchor to this system (see Figure 14c) and found that the ductility of the RC beam was further improved. This is because the additional expansion anchor provides an extra force transmission path, which maintains beam function after the Fe-SMA tendons are stripped from the adhesive material.

Nail-based mechanical anchorage system is an alternative method for the Fe-SMA NSMR system. As shown in Figure 14d, Fe-SMA strips can be easily fixed to the surface of the base concrete layer by the aid of direct-fasteners (e.g., nails) and nail-setting devices [131]. The total duration for installing and activating a 5-m Fe-SMA strip is within 20 min [132]. This method has been applied to some retrofitting cases in Switzerland [132]. However, nail-based anchorage systems may not be the best solution for bridges since the nails tend to loosen under HCF-loading conditions.

It should be noted that for RC structures, the target temperature during electric heating should be carefully controlled, since high temperatures may cause concrete cracking/damage and could be detrimental to the bond strength between Fe-SMA and concrete [73]. As reported in [125], a longitudinal splitting crack with a width of about 0.05 mm appeared in the mortar surface when the Fe-SMA was heated up to 190 °C. Most existing studies adopt a maximum activation temperature of around 160 °C, which can be regarded as a feasible target temperature.

#### 3.1.2. Strengthening for Steel Structures

**Figure 15:**
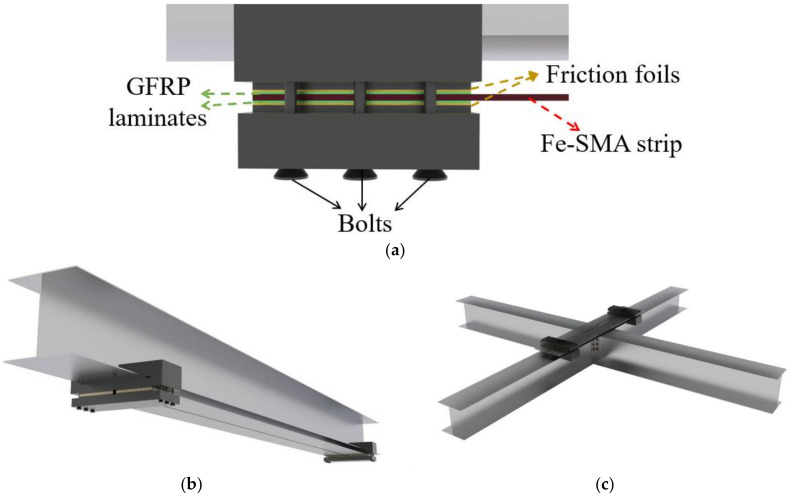
Schematic illustration of friction-based mechanical anchorage system for Fe-SMA strips in steel structures: (**a**) Configuration details; (**b**) Strengthening of metallic girders; and (**c**) strengthening of metallic connections.

The friction-based mechanical anchorage system, which is feasible for strengthening steel structures with Fe-SMA prestressing strips, was first developed by EMPA, as shown in Figure 15a [133]. Glass-fiber-reinforced plastic (GFRP) laminates and friction foils are also involved in this anchorage system, along with clamping plates and bolts which are necessary for anchoring. The GFRP laminates electrically insulate the Fe-SMA strips from the steel plate during the activation procedure, thus avoiding energy waste and reduction of heating efficiency. Extra friction foils are often used to increase the static friction coefficient for this joint. The experiment was first conducted on simple steel plates, and it was shown that a 2% pre-strain of the Fe-SMA strips can produce a recovery stress of about 330–410 MPa after heating to 260 °C, resulting in a compressive stress of about 35–74 MPa in the base steel plates. A fatigue test was further conducted and the results proved that the fatigue life of these strengthened steel plates was evidently increased and the propagation of initial cracks was postponed and even arrested in some cases [134]. Appropriate modifications were subsequently made and the applications were extended to fatigue strengthening of metallic girders (see Figure 15b) [135,136] and connections (see Figure 15c) [137]. Similar conclusions were drawn from these works.

Recently, a novel fatigue strengthening solution for steel structures using adhesively bonded Fe-SMA strips was investigated by EMPA. The adhesive Sika1277 was used to bond the Fe-SMA strips to the steel plates [138]. It is reported that the bonding force is approximately twice the prestress achieved in the Fe-SMA strips. No softening behavior was observed during the activation process, which means that the adhesive can securely anchor the Fe-SMA strip throughout the whole strengthening process [139]. Due to the bridging mechanism of the adhesive anchorage, crack opening in the base structure was suppressed and stress singularity at the crack tip was also significantly reduced [139]. However, future studies are still needed to investigate the time-varying behavior of this bonding-based anchorage system during the entire service life.

### 3.2. Seismic Dampers

**Figure 16:**
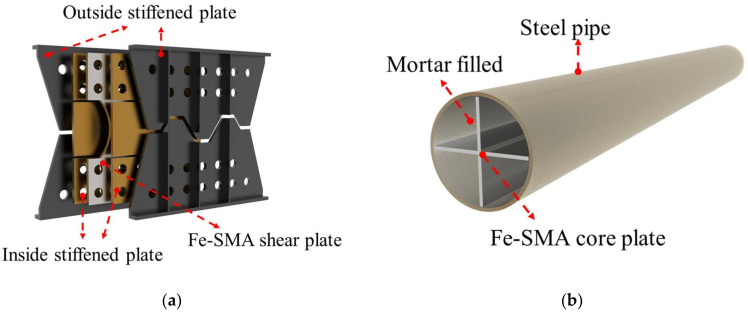
Configuration of Fe-SMA-based (**a**) BRS; and (**b**) BRB developed by a Japanese industry–academic–government joint research group.

Conventional metal dampers are usually made of steel with a reasonably low yield strength, which encourages early participation in energy dissipation. Ductility and durability are also important characteristics, since many strong ground motions followed by a series of aftershocks have been recorded in the past decades [140]. A Japanese industry–academic–government joint research group had developed Fe-SMA-based (Fe-15Mn-4Si-10Cr-8Ni) buckling restrained shear dampers (BRS, see Figure 16a) and buckling-restrained braces (BRB, see Figure 16b), and used them in the JP Tower Nagoya and The Aichi International Convention & Exhibition Center, Tokoname, respectively [28]. Related experiments have been conducted [23,28,75] and the results confirmed that the Fe-SMA seismic dampers exhibit considerably longer fatigue life (around ten times) than conventional steel dampers. Loading tests with random seismic wave inputs were also performed and the results showed that the seismic dampers exhibit stable energy absorption behavior under a wide range of deformation angles, reflecting a reliable performance during earthquake sequences [28].

**Figure 17:**
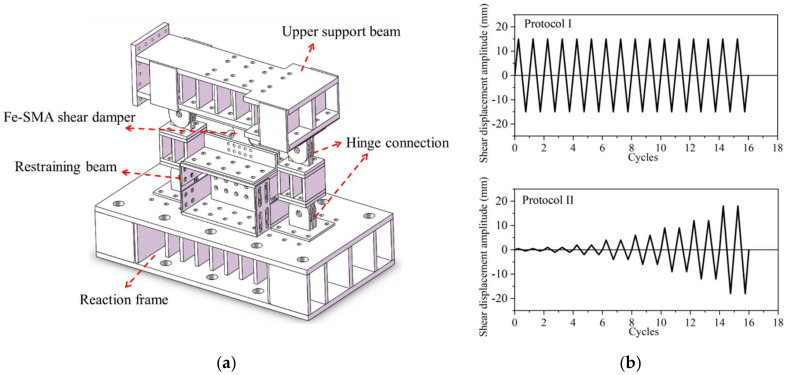
Laboratory experiment of Fe-SMA-based BRS: (**a**) illustration of test setup; and (**b**) loading protocols.

**Figure 18:**
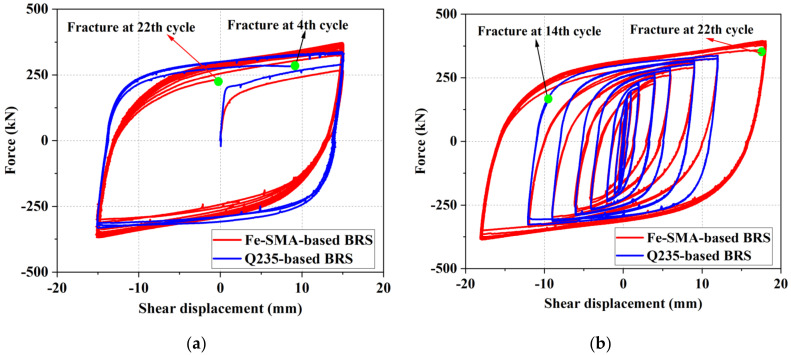
Hysteretic behavior for Fe-SMA- and Q235-based BRS under: (**a**) Protocol ‘I’; and (**b**) Protocol ‘II’.

**Figure 19:**
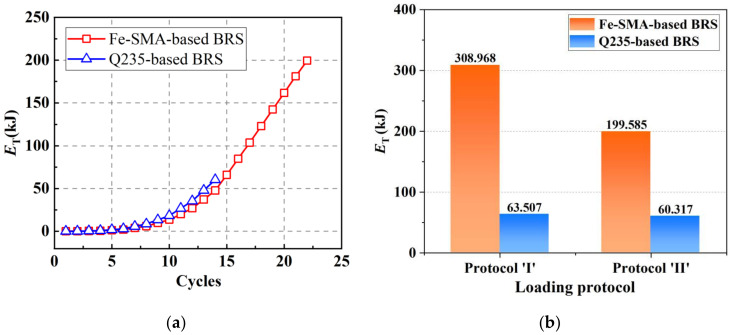
The total amount of accumulated energy dissipation (*E*_T_) for Fe-SMA- and Q235-based BRS: (**a**) *E*_T_ vs. Cycles under Protocol ‘I’; and (**b**) a comparison of *E*_T_ between these two metal BRS under different loading protocols.

**Figure 20:**
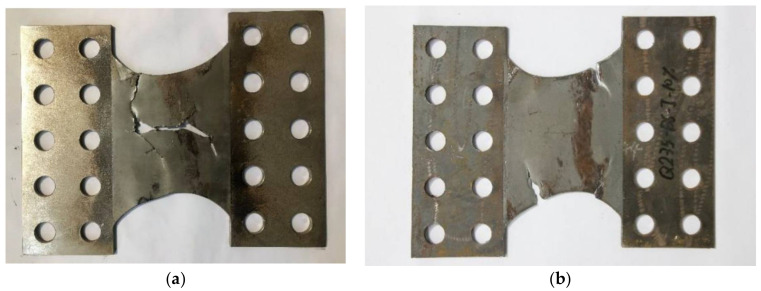
Macroscopic fracture behavior of (**a**) Fe-SMA-based BRS; and (**b**) Q235-based BRS under loading protocol ‘I’.

A more comprehensive experimental study on Fe-SMA (Fe-17Mn-5Si-10Cr-5Ni) BRS was conducted by the authors and co-workers recently [23] (Figure 17a). Loading protocols with constant and incremental symmetrical shear displacement amplitudes, marked as ‘protocol I’ and ‘protocol II’ in Figure 17b, respectively, were employed to investigate the hysteresis response of the Fe-SMA-based BRSs. Such loading protocols were also conducted on steel (Q235) BRSs with the same geometry, and the test results are compared in Figure 18. The hysteretic loops of Fe-SMA-based BRSs are slightly narrower than those of steel BRSs (half-life cycle EVD = 0.42 vs. 0.52 under constant displacement amplitude), which is consistent with the material-level observation described previously. Importantly, significantly enhanced fatigue resistance was achieved in the Fe-SMA-based BRSs, leading to a considerable increase in the total accumulated energy dissipation (*E*_T_) (Figure 19). Figure 20 shows the final crack patterns of Fe-SMA- and Q235-based BRSs in this experiment. The cracks of the Fe-SMA-based BRSs tended to be initiated in the center region of the core plates, with a subsequent crack propagation to the arc-shaped edge, whereas the cracks of the Q235-based ones were initiated from the arc-shaped edge region. Research opportunities exist in further investigating the reasons behind the difference in the fracture mechanism between Fe-SMA and steel shear dampers.

**Figure 21:**
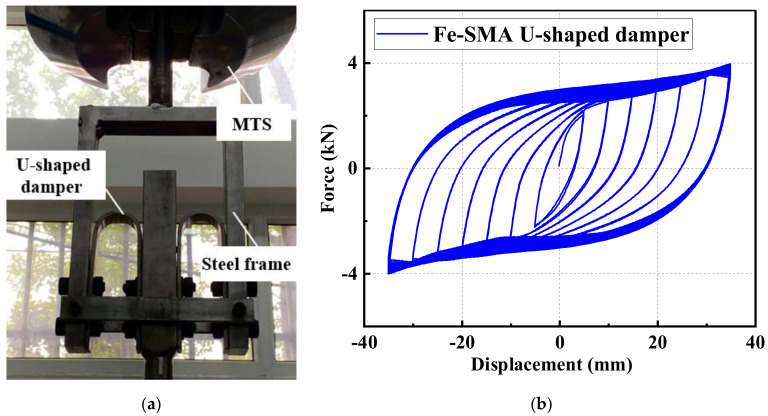
Fe-SMA U-shaped damper tests: (**a**) test setup; and (**b**) hysteretic response under incremental loading protocol.

More recently, the authors and co-workers have completed a series of tests on Fe-SMA U-shaped dampers, as shown in Figure 21a. It was found that the LCF life of the Fe-SMA dampers is 5–7 times that of their steel counterparts. A representative Fe-SMA damper hysteretic curve is shown in Figure 21b, where full and stable hysteresis curves are observed. The details of this experimental program will be published in a separate paper.

### 3.3. Advantages Compared with Alternative Solutions

Summarizing the above studies and applications, the main advantages of the Fe-SMA solutions are further elaborated here. In the field of retrofitting, the prestressing process of Fe-SMA strips is easier than that of the CFRP tendon-based reinforcement which currently prevails [133,136,139,141]. This is mainly because the former can be activated through electrical heating without any heavy hydraulic jacks or dedicated mechanical clamps. Moreover, the required fire protection for Fe-SMA strips can be less demanding than that required for CFRP strips. Studies have found that Fe-SMA strips have a positive effect in countering the relaxation behavior of structures at elevated temperatures [142]. Furthermore, corrosion, creep, and relaxation behavior under extreme environments (e.g., high-temperature and chlorine environments) have been investigated which confirmed the suitability and reliability of Fe-SMA [120,143,144,145,146,147,148,149,150]. Another attractive advantage of Fe-SMA-based strengthening strategy is its re-prestressing property, i.e., HCF-induced relaxation in recovery stress can be restored through repeated rounds of thermal activation [18,134,151]. This process is simple and can be implemented without the necessity for the time and labor-intensive disassembling procedure.

As for the Fe-SMA-based seismic dampers which have attracted great attention in the community of seismic designers, the fatigue-free and maintenance-free seismic design ambition may become possible. These significantly benefit society since the maintenance costs including those related to hazards have become an important part of governments’ expenditure over the years (for example, about 400 billion Euros are paid for maintenance of buildings in Europe [152]).

Despite the fact that the price of Fe-SMA is currently higher than conventional structural steel, the whole-life costing of Fe-SMA-based applications may be comparable with the conventional technologies. It is noted that Fe-SMA is much cheaper than NiTi SMA which is often criticized for its high cost [153,154,155,156,157]. Existing studies also found that the total costs of Fe-SMA- and CFRP-based strengthening solutions are comparable when the cost of dedicated mechanical clamps for prestressing CFRP strips as well as the cost of labor were considered [141]. Moreover, long term costs can be saved to some degree due to the low maintenance requirements and the corrosion-resistance property of Fe-SMA. Importantly, since Fe-SMA shares similar production process to stainless steel [9], it is optimistic to predict that the price of Fe-SMA has the potential to approach that of stainless steel as long as the demand matches with production quantities.

## 4. Further Research Needs

There is still a gap to be filled for a comprehensive understanding of Fe-SMA’s mechanical properties towards more confident application in practice. Metal plastic forming and heat treatment have a strong influence on the mechanical properties of Fe-SMA. Cold-worked Fe-SMA displays higher yield strength and higher shape recovery stress than that of hot-rolled ones [76], and the stress-strain curves may also show differences. The rationale behind the influence of these factors has not been comprehensively studied, and there are still limited constitutive models developed for Fe-SMA.

Welding is another important aspect for engineering application of Fe-SMA. Although the applicability of different welding technologies such as tungsten-inert gas, laser beam, and electron beam welding have been studied [158,159,160,161,162], some crucial welding properties and technologies are still under investigation. For example, in most of the existing experiments, Fe-SMA was welded to Fe-SMA or austenitic stainless steel in which the base metal shares the same metallography (γ-austenite). However, there has been little experimental research focusing on welding methods for connections between Fe-SMA and conventional structural steel. In addition, most of the existing studies focused on the effect of welding on the SME of Fe-SMA, whereas the mechanical properties of the weld itself and the heat-affected zone (HAZ) have been inadequately studied. Special attention should be paid to the fracture initiation mechanism of the weld, because the fatigue resistance as well as the plastic deformation capability of the weld and HAZ are usually inferior to the parent material of Fe-SMA, a case which leads to early fracture of the weld zone before the LCF failure of Fe-SMA. Further research opportunities exist in seeking for reliable weld techniques for Fe-SMA-based components, especially in the field of seismic engineering.

From a fracture mechanics’ point of view, previous research works have found that the crack distribution pattern of Fe-SMA-based dampers is very different from steel dampers [23], which may be related to the unique reversible martensitic transformation of Fe-SMA. However, an accurate explanation for the difference in the crack initiation and propagation behavior is still unavailable. Research opportunities exist in revealing how the microscopic feature of Fe-SMA would affect the macroscopic fatigue failure mode.

In future, Fe-SMAs may be used as next-generation structural steel due to its high strength, high ductility, excellent energy dissipation capability and LCF resistance during earthquakes. To this end, a more in-depth understanding of the mechanical behavior of Fe-SMA elements (such as springs, tendons, cables, etc.) and structural components (such as beams, columns, shear plate walls, etc.) is required. Since differences in mechanical properties exist between Fe-SMA and conventional structural steel, the applicability of the existing construction and design methods for normal steel to Fe-SMA-based structural components is questionable. In particular, a more unique system-level behavior is anticipated when the Fe-SMA elements are employed because of the special cyclic hardening and unloading spring-back behavior. Fe-SMA may also be used together with super-elastic Nitinol to reach a hybrid design which enables a good balance among self-centering capability, energy dissipation, and cost, compared with a pure Nitinol solution [44,163,164,165,166,167]. Preliminary seismic collapse safety assessment has revealed that the collapse capacity of beam-column joints equipped with Fe-SMA is significantly improved [168]. More studies are needed towards establishing a systematic and standardized design and construction process for Fe-SMA-based components and structural systems, and the entire design philosophy has to be revisited in future. Similarly, for the application of Fe-SMA in strengthening, further work is required to develop an integral design approach including the selection of materials, installation process, activation process, and quality check standard.

Last but not least, when one utilizes both the excellent LCF resistance and SME of Fe-SMA, a completely new structural design philosophy, namely, fatigue free and in situ recoverable structural design, may be developed. In other words, even if small residual deformation exists after an earthquake, a further deformation recovery may be promoted by heating the material via either electrical resistance or infrared heating, where the entire process is safe, quick, and economical. In this regard, the recoverable strain/recovery stress of cyclically ‘trained’ Fe-SMA (rather than that of the material under monotonic loading) is worth further studies.

## 5. Concluding Remarks

Fe-SMA is an emerging high-performance metal with unique properties that make it well suited to many applications in the construction sector. Its shape recovery properties result from reversible martensitic transformation and have been utilized for prestressing/retrofitting structural components. It is foreseeable that the convenient prestressing process and sound re-activating properties would promote the material for wider use in the field of structural retrofitting. A series of anchoring systems for Fe-SMA strengthening solution have been developed for different structural types.

The use of Fe-SMA as seismic-resistant material began in the 2000s. Satisfactory ductility and energy dissipation capability are identified. The repeated tension and compression-induced martensite, generated through cyclic loading, suppresses the micro-crack initiation and/or propagation which directly enhances the LCF life of Fe-SMA. These features make fatigue-free and maintenance-free seismic design philosophy possible in the near future.

Challenges and opportunities do remain for a more confident application of Fe-SMA in construction. For example, the influences of forming process and heating effects on the mechanical properties of Fe-SMA have not been fully investigated. Technologies for welding of Fe-SMA are needed. Constitutive models applicable to Fe-SMA should be proposed and the fundamental fracture mechanism of the material under different stress states needs to be clarified. Existing design approaches may be revisited when designing Fe-SMA-based structural components since many unique mechanical behaviors exist in Fe-SMA. The highly nonlinear stress-strain response, especially the substantial strain hardening characteristic may be a key design challenge for Fe-SMA-based seismic dampers. It is advised that more experiments on the component level and even system level should be conducted, seeking for a more comprehensive understanding of this material and its behavior in a structural system.

## Figures and Tables

**Table 1:** Physical properties for SMAs and conventional structural steels [5,11,12,13].

Property	Units	Value				
		Fe-SMA	NiTinol		Q235	SUS304
			(Martensite)	(Austenite)		
Density	g/cm^3^	7.2–7.5	6.45–6.5		7.86	7.93
Young’s modulus	GPa	170	28–41	83	201	193
Electrical resistivity	μΩ·cm	100–130	76–80	82–100	29.3	73
Specific Heat Capacity	J/kg·°C	540	836.8	836.8	745	500
Thermal conductivity	W/(m·°C)	8.4	8.6–10	18	61.1	16.3
Thermal expansion coefficient	(×10^−6^) °C^−1^	16.5	6.6	11	12.6	17.2
Melting point	°C	1320–1350	1240–1310		1468	1398–1454
Strain recovery limit	%	2	10		-	-
Poisson’s ratio	-	0.359	0.33		0.294	0.25

**Table 2:** Phase-transformation temperatures of SMAs.

Material	*M*_f_ (°C)	*M*_s_ (°C)	*A*_s_ (°C)	*A*_f_ (°C)	Ref.
Fe-SMA	−90	−75	85	110	[14]
Fe-SMA	−64	−60	103	162	[15]
NiTinol	−70–55	15–21	−22–2	17–30	[16]

**Table 3:** Mechanical properties of different metallic materials under quasi-static monotonic loading tests.

Material	*E*_0_/GPa	*f*_y_/MPa	*f*_u_/MPa	*f*_y_//*f*_u_	*ε*_u_	*f*_u2_/MPa	*ε*_u2_	*EL*(%)	Ref.
S30408	249	273	710	0.38	0.48	520	0.59	-	[22]
7A04-T6	72	537	594	0.91	0.08	590	0.10	-	[20]
LYP100	200	100	279	0.36	0.25	205	0.50	-	[19]
LY100	199	128	252	0.51	0.27	-	-	47.3	[25]
LY160	194	186	294	0.63	0.24	-	-	44.5	[25]
LY225	202	191	295	0.65	0.23	-	-	44.0	[25]
Q235	208	282	467	0.60	0.25	354	0.38	-	[24]
Q355	206	385	533	0.72	0.25	390	0.36	-	[24]
Q690	218	876	909	0.96	0.07	809	0.20	-	[24]
Fe-SMA	184	450	950	0.47	-	-	0.54	-	[26]
Fe-SMA	200	310	993	0.31	-	-	-	-	[27]
Fe-SMA	160	534	992	0.54	0.40	969	0.44	-	[21]
Fe-17Mn-5Si-10Cr-5Ni	172	297	774	0.38	0.45	698	0.48	-	[23]
Fe-28Mn-6Si-5Cr	170	250	800	0.31	0.40	-	-	-	[17]
Fe-15Mn-4Si-10Cr-8Ni	184	260	676	0.38	-	-	-	74.0	[28]

Notes: *E*_0_ refers to Young’s modulus; *f*_y_ refers to yield strength; f_u_ refers to ultimate tensile strength (UTS); *f*_u2_ refers to fracture stress; *ε*_u_ refers to the strain corresponds to UTS; *ε*_u2_ refers to the strain till fracture; *EL* refers to elongation after fracture.

**Table 4:** Recovery stresses for Fe-SMA under different activation conditions.

Ref.	Manufacturing Details	Specimen ^1^	Pre-Strain ^2^	Pre-Load	Activation Temperature	*f*_R_/MPa ^3^
[41]	Hot rolled at 1040 °C	SP-15 × 150	4%	50 MPa	160 °C	444
	Hot rolled at 1000 °C	SP-15 × 150	4%	50 MPa	160 °C	448
[14]	Hot rolled, solution treated at 1100 °C for 5 h, aging at 850 °C for 2 h	SP-0.7 × 1	4%	50 MPa	160 °C	330
		SP-0.7 × 1	4%	50 MPa	225 °C	380
[76]	Hot press at 1100 °C and cold rolled, solution treated at 1100 °C for 5 h and water quenched, aging at 850 °C for 2 h followed by air cooling	SP-0.7 × 1	4%	50 MPa	160 °C	565
[72]	Hot press at 1100 °C and cold rolled, solution treated at 1100 °C for 5 h and water quenched, aging at 850 °C for 2 h followed by air cooling	SP-0.8 × 2	2%	-	100 °C	290
		SP-0.8 × 2	4%	-	100 °C	303
		SP-0.8 × 2	2%	-	140 °C	317
		SP-0.8 × 2	4%	-	140 °C	355
[21]	Hot pressed and cold rolled	SP-1.5 × 15	0.5%	50 MPa	160 °C	293
		SP-1.5 × 15	2%	50 MPa	160 °C	346
		SP-1.5 × 15	2%	50 MPa	195 °C	388
		SP-1.5 × 15	4%	50 MPa	160 °C	334
		SP-0.5 × 15	4%	50 MPa	160 °C	331
		SP-1.5 × 15	6%	70 MPa	160 °C	298
		SP-1.5 × 15	8%	83 MPa	160 °C	334
[73]	Hot rolled at 1150 °C and cold rolled, solution treated at 1100 °C in H_2_N_2_-atmosphere for 5 h, aging at 850 °C for 2 h	SP-1.6 × 6	1%	50 MPa	200 °C	330
		SP-1.6 × 6	2%	50 MPa	200 °C	344
		SP-1.6 × 6	4%	50 MPa	200 °C	342
		SP-1.6 × 6	6%	50 MPa	200 °C	332
		SP-1.6 × 6	8%	50 MPa	200 °C	337
		SP-1.6 × 6	2%	50 MPa	349 °C	364
		SP-1.6 × 6	4%	50 MPa	350 °C	421
		SP-1.6 × 6	6%	50 MPa	350 °C	428
		SP-1.6 × 6	8%	50 MPa	350 °C	445

^1^ ‘SP’ refers to the sheet plate specimen, and is expressed in the form of ‘thickness × width’; ^2^ The pre-strain process is performed under room temperature environment; ^3^ ‘*f*_R_’ refers to the recovery stress of Fe-SMA.

**Table 5:** Numbers of cycles to failure of different metals.

Research Group	Material	Strain Amplitude					Ref.
		±1%	±3%	±5%	±7%	±9%	
Tongji University	Fe-17Mn-5Si-10Cr-5Ni	4007	880	210	102	83	[23]
Portland State University	GR345	536	69	27	16	-	[108]
	HPS485	400	51	21	13	-	[108]
	HS440	720	69	31	15	-	[108]
	LYP225	-	38	-	9	-	[108]
	LYP100	720	50	32	11	-	[108]
Imperial College	S355	495–732	53–107	22–24	9–15	-	[109]
	S235	439–521	16–21	8–20	3	-	[109]
	EN1.4301	266–335	27–78	7–61	2–4	-	[109]
Tsinghua University	LY100	512–694	82–103	-	-	-	[25]
	LY160	1008	121	40	-	-	[25]
	LY225	1220	101	46	-	-	[25]
Japanese National Institute for Materials Science	Fe-15Mn-(0-6)Si-10Cr-8Ni	2860–8500	-	-	-	-	[85]
	Fe-30Mn-(6-*x*)Si-*x*Al	2024–8070	-	-	-	-	[80]

## Data Availability

Data presented in this study are available in this article.
